# Effects of Vitamin D Supplementation on Blood Pressure in Patients With Type 1 Diabetes Mellitus: A Systematic Review of Clinical Trials

**DOI:** 10.1002/hsr2.70524

**Published:** 2025-03-02

**Authors:** Rasoul Ebrahimi, Mohammad Mahdi Masouri, Amir Abbas Salehi Amniyeh Khozani, Sana Mohammad Soltani, Seyed Aria Nejadghaderi

**Affiliations:** ^1^ School of Medicine Shahid Beheshti University of Medical Sciences Tehran Iran; ^2^ HIV/STI Surveillance Research Center, and WHO Collaborating Center for HIV Surveillance, Institute for Futures Studies in Health Kerman University of Medical Sciences Kerman Iran; ^3^ Systematic Review and Meta‐analysis Expert Group (SRMEG) Universal Scientific Education and Research Network (USERN) Tehran Iran

**Keywords:** blood pressure, diabetes mellitus, hypertension, systematic review, type 1, vitamin D

## Abstract

**Background and Aims:**

Hypertension exacerbates cardiovascular risks in patients with type 1 diabetes mellitus (T1DM), necessitating effective blood pressure (BP) management. Vitamin D deficiency is common in T1DM patients and is associated with an increased risk of cardiovascular diseases. This systematic review aimed to evaluate the impact of vitamin D supplementation on BP in T1DM patients.

**Methods:**

PubMed, Web of Science, Embase, Scopus, and Google Scholar were searched until March 2024. Clinical trials reported BP outcomes in patients with T1DM after vitamin D supplementation were included. Other types of studies and studies that did not report BP outcomes and those that had populations other than patients with T1DM were excluded. The National Institutes of Health (NIH) tool was used for the risk of bias assessment.

**Results:**

In total, eight studies, involving 328 participants, were included in this review. They were conducted between 2014 and 2024, with mostly conducted in Brazil (*n* = 5). While one study demonstrated a significant reduction in morning systolic and diastolic BP after vitamin D supplementation, five studies found no significant differences in systolic or diastolic BP. Another study noted a significant reduction in morning systolic and diastolic BP, with no significant changes in 24‐h ambulatory monitoring. Also, paricalcitol therapy did not significantly reduce systolic and diastolic ambulatory BP compared to placebo.

**Conclusion:**

The current evidence on the effect of vitamin D supplementation on blood pressure in patients with T1DM remains inconclusive. Nonetheless, more randomized controlled studies with larger sample sizes and longer follow‐up durations are essential to establish the association between vitamin D and BP in this population.

## 1 Introduction

Type 1 diabetes mellitus (T1DM) is a lifelong autoimmune disorder in which the immune system attacks and destroys the insulin‐producing beta cells in the pancreas, resulting in a permanent reliance on external insulin administration. The global prevalence of T1DM is increasing, with significant implications for public health. It is estimated that the incidence of T1DM is rising worldwide, contributing to a substantial burden of disease [1]. T1DM is a major cardiovascular risk factor, contributing to increased morbidity and mortality due to its association with various cardiovascular diseases [2].

Hypertension is a common comorbidity in patients with T1DM and significantly exacerbates the risk of cardiovascular events. Elevated blood pressure (BP) in patients with T1DM is a critical factor that accelerates the development of atherosclerosis and other cardiovascular complications, thereby increasing the overall cardiovascular risk [3]. Effective management of BP is essential to reduce the incidence of cardiovascular morbidity and mortality in this population [4].

Vitamin D, a fat‐soluble secosteroid hormone, is crucial for calcium and phosphorus metabolism and has various extraskeletal effects, including immune modulation and cardiovascular health [5]. Hypovitaminosis D is prevalent in patients with autoimmune diseases such as Crohn's disease, type 2 diabetes mellitus, rheumatoid arthritis, lupus, multiple sclerosis, and certain cancers like colon, prostate, breast, and pancreatic cancer. This deficiency may exacerbate inflammatory responses, worsening insulin resistance in affected individuals [6, 7, 8, 9, 10]. Vitamin D plays an immunomodulatory role by affecting immune system cells through cytokine production [11]. Research indicates that individuals with vitamin D deficiency experience a decreased risk of upper respiratory infections following supplementation. Additionally, patients with asthma or respiratory diseases benefit from vitamin D, as it lowers the incidence of acute respiratory infections and enhances lung function. In autoimmune diseases, vitamin D deficiency may exacerbate conditions due to its regulatory effects on the adaptive immune system. Specifically, the lack of calcitriol can lead to decreased regulation, which increases susceptibility to autoimmune disorders among affected individuals [12]. Vitamin D also reduces oxidative stress by enhancing cellular glutathione and superoxide dismutase levels [11]. Low vitamin D levels are linked to increased oxidative stress markers, correlating with common carotid intima thickening, a sign of cardiovascular dysfunction. Conversely, individuals with adequate serum 25(OH)D levels, particularly those consuming calcium and vitamin D through dairy products, are at a lower risk for arterial hypertension. The detrimental effects of vitamin D deficiency on the renin‐angiotensin‐aldosterone system (RAAS) activation and endothelial function are proposed mechanisms for this increased risk of arterial hypertension [7, 13, 14]. However, the exact mechanisms and effects of vitamin D on human RAAS and BP regulation remain unclear based on current research [15]. Vitamin D deficiency is prevalent among T1DM patients and has been associated with poor glycemic control and increased risk of diabetes‐related complications, such as cardiovascular diseases [16].

Recent studies have explored the potential benefits of vitamin D supplementation in managing BP among patients with T1DM. For instance, a clinical trial demonstrated that high‐dose vitamin D supplementation significantly reduced both systolic BP (SBP) and diastolic BP (DBP) in normotensive T1DM patients [17]. Another study found improvements in cardiovascular autonomic neuropathy parameters with high‐dose vitamin D supplementation in T1DM patients, although no significant changes in BP were observed [18]. The relationship between vitamin D and glycemic control has also been investigated. In this regard, evidence showed that vitamin D deficiency is associated with higher glycated hemoglobin (HbA1c) levels and poorer glycemic control in diabetic patients, suggesting that vitamin D supplementation could potentially improve glucose homeostasis [19]. However, the evidence remains inconclusive, with some studies showing no significant effects on BP or glycemic control [18, 20]. Previous systematic reviews have examined the effect of vitamin D supplementation on glycemic control and other outcomes, including maternal and neonatal outcomes, in patients with type 2 and gestational diabetes mellitus [21, 22]. Due to inconsistencies in findings regarding the influence of vitamin D on BP in patients with T1DM, there is a need for a comprehensive assessment of the association. Therefore, this systematic review aimed to evaluate the effects of vitamin D supplementation on BP in patients with T1DM.

## 2 Methods

We followed the recommendations outlined in the Preferred Reporting Items for Systematic Reviews and Meta‐Analyses (PRISMA) guidelines [23].

### 2.1 Search Strategy

We carried out searches on the PubMed, Web of Science, Embase, and Scopus databases until March 28, 2024, without any limitations on language, date, or type of article. We used terms related to “Vitamin D,” “Diabetes,” and “Clinical Trial,” including (“Type 1 Diabetes Mellitus” OR “Insulin Dependent Diabetes Mellitus” OR “Juvenile Onset Diabetes Mellitus” OR “IDDM” OR “diabetes*”) AND (“Vitamin D*” OR “Cholecalciferol*” OR “Dihydroxycholecalciferol” OR “Ergocalciferols” OR “Calcitriol”) AND (“Clinical Trial” OR “Randomized Controlled Trial” OR “RCT”). After the full‐text review, we evaluated the 300 first results of the Google Scholar search engine up to April 24, 2024, as gray literature search [24] (Supporting Information S1: Table 1). Moreover, backward and forward citation searching for the included studies was performed.

### 2.2 Study Selection

We included articles on participants diagnosed with T1DM, evaluated the effects of vitamin D supplementation as the intervention, reported the occurrence of hypertension or changes in BP, and those provided measures of effects like odds ratio or risk ratio or provided the necessary raw data to calculate these measures. Only clinical trials or randomized controlled trials were eligible.

The exclusion criteria were (1) studies that included patients with types of diabetes other than T1DM, like gestational diabetes or T2DM; (2) studies that reported on the levels or effects of other vitamins, minerals, or supplements besides vitamin D or did not use vitamin D supplementation; (3) studies that did not report BP alterations or the occurrence of hypertension; (4) other study designs, such as in vitro and in vivo studies, observational studies, animal studies, case reports or series, review articles, notes, and meta‐analyses. If other supplements or minerals were used, only data specifically reported for vitamin D were considered. Other reported outcomes of the eligible studies were provided as the secondary outcomes. Also, if primary studies included both T1DM and T2DM, only data specifically reported for T1DM were considered.

Two independent reviewers screened the titles and abstracts of the identified studies. Full‐text articles were retrieved for those that met the inclusion criteria in the screening stage. Discrepancies were resolved through discussion or consultation with the principal investigator.

### 2.3 Data Extraction

A Microsoft Excel Office, version 2019 data extraction sheet was designed by the senior investigator to collect data. The two authors independently gathered the information, and any disagreements were resolved by a third author. The following study characteristics were extracted: the first author's name, year of publication, country where the study was conducted, as well as the clinical trial phase and blinding. Additionally, information on patient characteristics was collected, including age, sex, duration of diabetes mellitus, comorbidities, habitual history, angiotensin‐converting enzyme inhibitors or angiotensin II receptor blockers (ACEI/ARB) use, body mass index (BMI), and ethnicity. Dosage and duration of vitamin D supplementation, BP outcomes, and clinical and laboratory data (including HbA1c, fasting plasma glucose [FPG], total, daily and prandial insulin dose, total cholesterol, high‐density lipoprotein [HDL], low‐density lipoprotein [LDL], triglyceride, creatinine, glomerular filtration rate [GFR] and heart rate) before and after vitamin D supplementation were extracted.

### 2.4 Quality Assessment

To assess the quality of each study included, two authors independently evaluated them and any disagreements were resolved through discussion. The quality assessment tool of the National Institutes of Health (NIH) for controlled intervention studies and case series was utilized [25]. To summarize, the components for uncontrolled studies consist of: a clear and concise research question, detailed information about the study population, inclusion of consecutive cases, comparability between subjects, a detailed description of the intervention, well‐established outcome measures, sufficient follow‐up duration, suitable statistical methods, and a clear explanation of the results.

For controlled intervention studies, 14 domains were taken into account, including the randomization of the study, concealing of the treatment allocation, whether or not participants, providers, and assessors were blinded, similarity of the groups at baseline, the rate of drop‐outs, adherence to intervention, avoiding other interventions, validating the outcome measures used, sufficiency of the sample size, and pre‐specifying outcomes and subgroups. Studies were categorized as poor, fair, or good based on their quality score. For uncontrolled studies, scores of 0–3, 4–6, and 7–9 were deemed poor, fair, and good, respectively. For controlled studies, scores of 0–6, 7–10, and 11–14 were considered poor, fair, and good respectively.

## 3 Results

### 3.1 Study Selection

**Figure 1:**
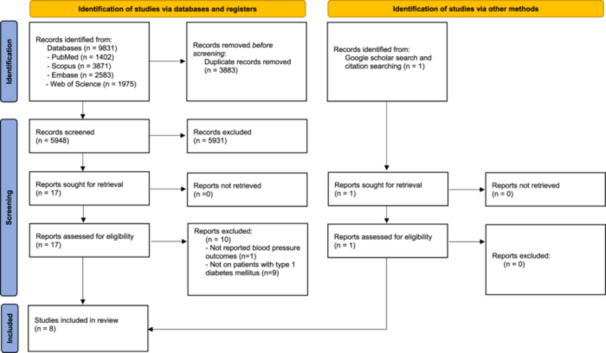
Study selection process for the articles in the systematic review.

Initially, 9831 articles were found through a systematic search. After eliminating duplicate results, 5948 studies were screened through title and abstract. Among them, 5931 studies were excluded from the screening process. Following this, 17 studies were examined in the full‐text review. Nine studies were excluded since they were not conducted on patients with T1DM and one was excluded because of not reporting relevant outcomes. So, seven studies were included in this step [17, 18, 26, 27, 28, 29, 30]. One additional study was included by citation searching and Google Scholar search [31]. Finally, a total of eight studies were included in this systematic review [17, 18, 26, 27, 28, 29, 30, 31] (Figure 1).

### 3.2 Study Characteristics

Baseline characteristics of the included studies are provided in Table 1. Five studies were conducted in Brazil [17, 18, 26, 28, 31], one study in each of the countries Denmark [29], Canada [27], and the United States [30]. The studies were conducted between 2014 [29] and 2024 [28]. There were five pilot trials [17, 18, 26, 28, 31] and the phases of other studies were not specified [27, 29, 30]. Two studies had a control group and were double‐blind [29, 30]. Various doses of vitamin D were used, ranging from 4000 international unit (IU)/day to 50,000 IU/week, depending on baseline vitamin D levels of the patients [18, 26, 28]. The total number of participants was 328 with an age range between 10 and 74 years. Males made up 53% (*n* = 174) of the total participants. The duration of diabetes ranged from 6 [27] to 66 [29] years. Furthermore, 27 participants were known to have hypertension [18, 26, 28], 48 had dyslipidemia [17, 18, 26, 28], 126 had diabetic nephropathy [17, 18, 26, 28, 29, 31], 54 had peripheral neuropathy [17, 18, 26, 28], and 34 had retinopathy [17, 18, 26, 28]. Additionally, among the participants, 39 were smokers [27, 30, 31] and 43 had a history of alcohol consumption [17, 18, 28]. Forty‐eight participants had previously used ACEI/ARB medication [17, 18, 26, 28]. Also, 27 were from a white ethnicity background [30], 48 were white European [29], and 23 were non‐Caucasian [27] (Table 1).

**Table 1:** Study and patient characteristics.

Study ID	HbA1c (%)	FPG (mg/dL)	Total daily insulin dose (IU)	Daily dose of basal insulin (IU)	Prandial insulin dose (IU)	BMI (kg/m^2^)	Total cholesterol (mg/dL)	HDL (mg/dL)	Non‐HDL (mg/dL)	LDL (mg/dL)	TG (mg/dL)	Creatinine (mg/dL)	GFR (mL/min/1.73 m^2^)	Heart rate (bpm)
Felício et al. 2017 [31]	Before VD: 9.3 ± 2.7 After DV: 9.3 ± 2.3 P = NS	SDG before VD, median (minimum– maximum): 62.5 [29, 31, 32, 33, 34, 35, 36, 37, 38, 39, 40, 41, 42, 43, 44, 45, 46, 47, 48, 49, 50, 51, 52, 53, 54, 55, 56, 57, 58, 59, 60, 61, 62, 63, 64, 65, 66, 67, 68, 69, 70, 71, 72, 73, 74, 75, 76, 77, 78, 79, 80, 81, 82, 83, 84, 85, 86, 87, 88, 89, 90, 91, 92, 93, 94, 95] SDG after VD: 66.5 (33–111) P = NS	Before VD: 65.2 ± 29.9 After VD: 62.4 ± 27.1 P = NS	Before VD: 44.2 ± 20.1 After VD: 42.6 ± 19.2 P = NS	Before VD: 22.0 ± 12.6 After VD: 20.8 ± 11.9 P = NS	Before VD: 25.7 ± 3.4 After VD: 25.6 ± 3.5 P = NS	NA	NA	NA	NA	NA	Before VD: 0.94 ± 0.15 After VD: 0.98 ± 0.18 P = NS	Before VD: 108.6 ± 23.2 After VD: 106.5 ± 24.1 P = NS	NA
Joergensen et al. 2014 [29]	Before VD: 8.6 ± 0.8 After VD: NA	NA	NA	NA	NA	BMI before VD: 28 ± 5 kg/m2 After VD: NA	Before VD: 4.5 ± 0.9 mmol/L After VD: NA	NA	NA	NA	NA	Plasma creatinine before VD: 141 ± 50 lmol/l After VD: NA	Before placebo: 46 ± 16 After placebo: 46 ± 15 Before paricalcitol: 44 ± 16 After paricalcitol: 41 ± 16	NA
Deda et al. 2017 [27]	Before VD: 9.0 ± 1.5 After VD: 8.9 ± 1.7 *p* = 0.4	Before VD: 10.6 ± 3.7 mmol/L After VD: 9.5 ± 4.6 mmol/L *p* = 0.3	NA	NA	NA	Before VD: 23.7 ± 4.0 After VD: 23.8 ± 3.9 *p* = 0.6	Before VD: 4.4 ± 0.7 mmol/L After VD: 4.3 ± 0.7 mmol/L *p* = 0.5	Before VD: 1.5 ± 0.5 mmol/L After VD: 1.4 ± 0.4 mmol/L	NA	Before VD: 2.4 ± 0.6 mmol/L After VD: 2.4 ± 0.7 mmol/L	Before VD: 0.9 ± 0.6 mmol/L After VD: 1.0 ± 0.8 mmol/L	ACR before VD: 1.0 ± 1.2 mg/mmol After VD: 1.2 ± 2.8 mg/mmol	eGFR, before VD: 109.4 ± 19.8 mL/min per 1.73 m2 eGFR, after VD: 108.8 ± 19.64 mL/min per 1.73 m2	NA
Felício et al. 2024 [28]	Before VD: 9.5 ± 2.3 After VD: 9.7 ± 2.6 P = NS(0.92)	Before VD: 165 ± 94 After VD: 179 ± 101 *p* = 0.41	NA	Before VD: 35 ± 17 After VD: 36 ± 17 P = NS (0.93)	Before VD: 22 ± 11 After VD: 23 ± 11 *p* = 0.60	Before VD: 24 ± 4 After VD: 24 ± 4 P = NS (0.94)	Before VD: 171 ± 40 After VD: 180 ± 59 *p* = 0.99	Before VD: 51 ± 37 After VD: 44 ± 10 *p* = 0.26	NA	Before VD: 103 ± 30 After VD: 108 ± 48 *p* = 0.84	Before VD: 99 ± 56 After VD: 108 ± 70 *p* = 0.65	Before VD: 0.8 ± 0.2 After VD: 0.8 ± 0.2 *p* = 0.61	NA	Before VD: 83 ± 13 After VD: 82 ± 14 *p* = 0.73
de Queiroz et al. 2021 [17]	Before VD: 10 ± 3 After VD: 10 ± 3 P = NS	Before VD: 171 ± 79 After VD: 181 ± 99 P = NS	NA	Before VD: 33 ± 13 After VD: 32 ± 12 P = NS	Before VD: 22 ± 12 After VD: 23 ± 13 P = NS	Before VD: 24 ± 4 After VD: 23 ± 3 P = NS	Before VD: 164 ± 40 After VD: 162 ± 50 P = NS	Before VD: 48 ± 14 After VD: 46 ± 12 P = NS	NA	Before VD: 104 ± 31 After VD: 103 ± 31 P = NS	Before VD: 98 ± 47 After VD: 115 ± 83 P = NS	Before VD: 0.8 ± 0.2 After VD: 0.8 ± 0.2 P = NS	Before VD: 115 ± 25 After VD: 111 ± 24 P = NS	Before VD: 80 ± 14 After VD: 78 ± 14 P = NS
Silva et al. 2020 [18]	Before VD: 9.5 ± 2.3 After VD: 9.6 ± 2.5 *p* = 0.153	Before VD: 168 ± 94 After VD: 173 ± 95 *p* = 0.951	Before VD: 57 ± 27 After VD: 58 ± 27 *p* = 0.682	Before VD: 36 ± 17 After VD: 36 ± 18 *p* = 0.193	Before VD: 22 ± 11 After VD: 23 ± 12 *p* = 0.563	Before VD: 24.0 ± 4.3 After VD: 24.0 ± 4.5 *p* = 0.674	Before VD: 173 ± 40 After VD: 180 ± 60 *p* = 0.253	Before VD: 52 ± 38 After VD: 44 ± 11 *p* = 0.342	Before VD: 129 ± 32 After VD: 128 ± 48 *p* = 0.318	Before VD: 104 ± 30 After VD: 107 ± 48 *p* = 0.609	Before VD: 118 ± 44 After VD: 129 ± 96 *p* = 0.57	Before VD: 0.8 ± 0.3 After VD: 0.8 ± 0.25 *p* = 0.381	NA	Before VD: 83.5 ± 14 After VD: 83 ± 14 *p* = 0.416
Nwosu et al. 2022 [30]	Before VD: 7.62 ± 1.35 After VD: 7.47 ± 1.69 *p* = 0.77	Before VD: 125.83 ± 25.00 After VD: 111.13 ± 35.78 *p* = 0.18	Before VD: 37.00 ± 29.61 After VD: 27.17 ± 14.41 *p* = 0.23	NA	NA	Before VD: 22.03 ± 5.41 After VD: 22.01 ± 4.15 *p* = 0.99	NA	NA	NA	NA	NA	NA	NA	NA
de Souza et al. 2022 [26]	Before VD: 9.6 ± 2 After VD: 9.8 ± 3 P = NS (0.250)	Before VD: 168 ± 96 After VD: 80.6 ± 103 *p* = 0.497	Before VD: 55.8 ± 27 After VD: 58.1 ± 27 *p* = 0.708	Before VD: 35.6 ± 17 After VD: 36 ± 18 *p* = 0.957	Before VD: 22.3 ± 12 After VD: 23.3 ± 12 *p* = 0.177	Before VD: 24 ± 4 After VD: 24 ± 4 P = NS (0.247)	Before VD: 171.7 ± 41 After VD: 177 ± 54 *p* = 0.292	Before VD: 52 ± 38 After VD: 44.6 ± 11 *p* = 0.293	Before VD: 123.5 ± 38 After VD: 125.7 ± 50 P = NS (0.289)	Before VD: 103.7 ± 31 After VD: 107.9 ± 49 *p* = 0.714	Before VD: 97.7 ± 51 After VD: 109.6 ± 70 *p* = 0.186	NA	Before VD: 120 ± 37 After VD: 117 ± 36 *p* = 0.247	NA

*Note:* The values represent the mean and standard deviation.

Abbreviations: ACR, albumin creatinine ratio; bpm, beat per minute; BMI, body mass index; eGFR, estimated glomerular filtration rate; FPG, fasting plasma glucose; HDL, high‐density lipoprotein; IU, international unit; LDL, low‐density lipoprotein; NS, not significant; NA, not available; SDG, standard deviation of glucose; TG, triglyceride; VD, vitamin D.

### 3.3 Systolic and Diastolic BP

Of the studies included, six did not report a significant change in either systolic or diastolic BP levels following vitamin D supplementation [18, 26, 27, 29, 30, 31]. However, one study found a significant reduction in morning systolic and diastolic BP was observed based on 24‐h ambulatory BP monitoring (ABPM) [17]. Additionally, another study reported a significant decrease in total, awake, and morning SBP among participants with cardiovascular autonomic neuropathy (CAN) after supplementation with vitamin D, including a notable drop in morning BP surge [28].

The study by De Souza et al. reported no significant changes in DBP after vitamin D supplementation (pre‐supplementation mean: 71 ± 9 mmHg, post‐supplementation mean: 72 ± 9 mmHg, *p* = 0.153) [26]. Similarly, the study by Nwosu et al. found no significant differences in both DBP and SBP between the placebo and ergocalciferol groups over the 12‐month study period. The mean DBP and SBP levels for both groups remained stable over time and did not significantly differ from baseline (*p* = 0.64). At 12 months, the SBP in the placebo and the ergocalciferol group were 109.1 ± 10.4 and 107.3 ± 12.6, respectively (*p* = 0.84). The baseline DBP for both groups was not significantly different (*p* = 0.98). At 12 months, the DBP in the placebo and the ergocalciferol group were 69.6 ± 10.3 and 66.3 ± 11.3, respectively (*p* = 0.49) [30].

However, De Queiroz et al. noted a significant reduction in morning systolic and diastolic BPs with vitamin D supplementation, as measured by 24‐h ABPM. Although, the other 24‐h ABPM parameters remained stable. The reduction in morning DBP showed a correlation with the final vitamin D values. Moreover, there was a trend towards a reduction in the sleep‐through morning BP surge [17]. In contrast, Deda et al. showed that there were no significant changes in either systolic or diastolic BP levels after a follow‐up period of 4.8 months. The mean SBP and SBP z‐score slightly increased from baseline, but the change was not statistically significant. Similarly, there were no significant changes in mean DBP or DBP z‐score after the follow‐up period (SBP at baseline and after vitamin D supplementation: 113.5 ± 11.6 and 115.9 ± 11.0, respectively (*p* = 0.30); DBP at baseline and after vitamin D supplementation: 64.42 ± 6.6 and 67.0 ± 8.8, respectively (*p* = 0.20)) [27]. Similarly, Felicio et al. reported no significant changes in SBP or DBP after the intervention period. The mean SBP and DBP levels during both vigil and sleep periods remained stable and nonsignificant from the initial to the final visit. Additionally, the standard deviation of SBP and DBP during sleep did not significantly change after the intervention period [31].

Data from the study by Silva et al. showed that there were no significant changes in SBP and DBP levels in T1DM patients with CAN who received vitamin D supplementation. The mean SBP levels remained stable and nonsignificant from before to after vitamin D supplementation. Similarly, there were no significant changes in mean DBP levels before and after the intervention period [18]. In the study conducted by Felicio et al. baseline measurements revealed that patients with CAN exhibited higher systolic sleep BP (115 ± 14 vs. 107 ± 12 mmHg, *p* = 0.04) and reduced nocturnal dipping in both systolic (3 ± 5 vs. 8 ± 6 mmHg, *p* = 0.009) and diastolic blood pressure (6 ± 7 vs. 11 ± 8 mmHg, *p* = 0.019) compared to the control group. Following vitamin D supplementation, the CAN group had a significant reduction in total awake SBP (118 ± 13 vs. 115 ± 12 mmHg, *p* = 0.006), morning SBP (120 ± 20 vs. 114 ± 17 mmHg, *p* = 0.038), and morning BP surge (13 ± 13 vs. 5 ± 14 mmHg, *p* = 0.04). However, there was no improvement in systolic nocturnal dipping in these patients. In contrast, no changes were observed in the parameters analyzed by ABPM after vitamin D supplementation in the control group without CAN [31. Joergensen et al. showed that paricalcitol therapy did not significantly reduce systolic ambulatory BP compared to placebo. The mean systolic ambulatory BP was reduced with a mean (standard deviation) of one mmHg during paricalcitol therapy, while it increased by two mmHg during placebo therapy, although the difference was not statistically significant (*p* = 0.53). Furthermore, diastolic ambulatory BP remained unchanged during paricalcitol therapy, while it was reduced by two mmHg during placebo therapy, which was not statistically significant (*p* = 0.48) [29] (Table 2).

**Table 2:** Dosage of vitamin D supplementation and BP outcomes.

Study ID	De Souza et al. 2022 [26]	Nwosu et al. 2022 [30]	De Queiroz et al. 2021 [17]	Deda et al. 2017 [27]	Felicio et al. 2017 [31]	Silva et al. 2020 [18]	Felicio et al. 2024 [28]	Joergensen et al. 2014 [29]
Vitamin D dosage	– Basal VD levels between 30 and 60 ng/mL: 4000 IU/day of cholecalciferol – Basal VD levels below 30 ng/mL: 10,000 IU/day of cholecalciferol	– Ergocalciferol 50,000 IU orally once weekly for 2 months. – Dosage was reduced to once every other week for 10 months after 2 months.	– Supplementation of 10,000 IU/day for 3 consecutive months for 25(OH)D levels below 30 ng/mL – 4000 IU/day of vitamin D for 25(OH)D levels between 30 and 60 ng/mL, aiming to maintain serum levels between 30 ng/mL and 100 ng/mL – Cholecalciferol used for supplementation, with each drop containing 500 IU.	– Dosing regimen for oral Ddrops (D3) varied based on baseline 25‐OH‐VitD levels.	– Supplementation of 10,000 IU/day for 3 months for VD insufficiency and deficiency (serum 25(OH)D < 30 ng/mL) – 4000 IU/day of VD for 25(OH)D levels between 30 and 60 ng/mL to keep serum levels above 30 ng/mL and less than 100 ng/mL – Cholecalciferol supplementation: 1 mL = 20 drops = 4,000 IU.	– Supplementation with 4,000 IU/day of cholecalciferol for individuals with values ≥ 30 ng/dL – 10,000 IU/day of VD supplementation for a duration of 12 weeks for those with levels < 30 ng/dL	– 4000 IU/day cholecalciferol for those with 25(OH)D levels of 30‐60 ng/mL – 10,000 IU/day for those with deficiency and/or insufficiency (< 30 ng/mL).	– Starting dose for paricalcitol: 1 µg per day; 2 µg per day if PTH levels > 53 pmol/l (< 500 pg/ml) – Dose adjustments based on PTH, ionized calcium, and phosphate levels at 2 and 6 weeks – Participants titrated up to 2 µg per day, maintained at 1 µg per day, or downregulated for hypercalcemia/elecated calcium phosphate – If already on 1 µg daily, dose reduced to 1 µg three times weekly
Duration	12 weeks	12 months	12 weeks	– Treatment duration: 12 weeks. – 25‐OH‐VitD reassessed at the end of 12 weeks; If still insufficient, treatment continued for another 12 weeks.	12 weeks	12 weeks	12 weeks	12 weeks
Goal	By the end of the trial endpoint: VD levels between 30 and 60 ng/mL	Maintain serum 25(OH)D between 20 and 100 ng/dL Early increase in serum 25(OH)D concentration is desired.	Reaching and maintaining levels above 30 ng/mL	NA	Achieve and maintain levels above 30 ng/mL	NA	Maintain serum levels above 30 ng/mL and below 100 ng/mL	NA
BP outcome	– SBP: before VD: 115 ± 11 mmHg; After VD: 115 ± 12 mmHg; *p* = 0.765 – DBP: before VD: 71 ± 9 mmHg; after VD: 72 ± 9; *p* = 0.153	– No significant effect of ergocalciferol on systolic or diastolic BP observed.	– A significant decrease in morning systolic and diastolic BP based on 24‐h ABPM was found. – Other 24‐h ABPM parameters remained unchanged. – Final vitamin D levels were negatively correlated with reduction in morning diastolic BP (*r* = −0.04, *p* < 0.05). – A trend towards a decrease was observed in sleep‐through morning BP surge (16 ± 16 vs. 8 ± 17, *p* < 0.05).	– No significant changes observed in SBP or DBP – No significant changes observed in the z‐scores for SBP or DBP.	– No significant changes detected in vigil systolic or diastolic BP, sleep systolic or diastolic BP, or SD of sleep systolic or diastolic BP.	– SBP: before VD: 114 ± 15 mmHg; after VD: 112 ± 15 mmHg; *p* = 0.589 – DBP: before VD: 70 ± 11 mmHg; after VD: 69 ± 11 mmHg; *p* = 0.711	– The CAN patient group had higher systolic sleep BP and lower nocturnal dipping than the control group at baseline. – Following VD supplementation, the CAN group experienced a significant reduction in total, awake, and morning SBP levels, along with a drop in the morning BP surge. – No improvement in systolic nocturnal dipping was observed in the CAN patient group – There were no changes in the parameters analyzed by ABPM after VD supplementation in the control group	– Reduction of Systolic ambulatory BP during paricalcitol therapy: 1 ± 20 mmHg – Increase in systolic ambulatory BP during placebo therapy: 1 ± 20 mmHg – (*p* = 0.53) ○ Diastolic ambulatory BP showed no significant change during paricalcitol therapy. – Reduction in diastolic ambulatory BP during placebo therapy: 2 ± 9 mmHg; *p* = 0.48

*Note:* The values are mean ± standard deviation.

Abbreviations: ABPM, ambulatory BP monitoring; BP, blood pressure; CAN, cardiovascular autonomic neuropathy; DBP, diastolic BP; IU: international unit; SBP, systolic BP; SD, standard deviation; VD, vitamin D.

### 3.4 Clinical and Laboratory Measurements

HbA1c levels, FPG levels, and insulin doses remained unchanged in five studies [17, 26, 28, 30, 31]. Deda et al. reported a nonsignificant decrease in HbA1c levels and FPG levels after vitamin D supplementation [27]. Silva et al. found a nonsignificant decrease in HbA1c levels and a nonsignificant increase in FPG levels [18]. No significant changes were reported in insulin doses in any study (Table 1).

In terms of BMI and lipid parameters, some studies reported differences before and after vitamin D supplementation [17, 18, 27, 28, 29, 31]. Deda et al. observed a nonsignificant increase in BMI after vitamin D supplementation, along with a nonsignificant decrease in total cholesterol [27].

Regarding creatinine, GFR, and heart rate, five studies reported no significant changes before and after vitamin D supplementation [17, 18, 28, 30, 31]. Deda et al. found no significant change in heart rate, but a nonsignificant increase in the albumin creatinine ratio and a nonsignificant decrease in estimated GFR [27]. Joergensen et al. found a significant increase in estimated GFR after paricalcitol supplementation [29]. De Souza et al. reported no data on creatinine levels but found no significant differences in estimated GFR or heart rate before and after vitamin D supplementation [26] (Table 1).

### 3.5 Quality Assessment

All six noncontrolled studies were rated as good quality [17, 18, 26, 27, 28, 31] (Supporting Information S1: Table 2). Among controlled interventional studies, Nwosu et al. met all quality criteria and had an overall quality score of 14 [30]. Joergensen et al. met all but two quality criteria and had an overall quality score of 12. It did not fully meet the quality criteria regarding treatment allocation concealment and sufficiency of sample size [29] (Supporting Information S1: Table 3).

## 4 Discussion

We found that most studies reported no significant effects of vitamin D supplementation on BP in T1DM, despite some significant reductions in BP were reported in some studies. We also found that most studies showed no significant changes in HbA1c levels, FPG levels, insulin doses, BMI, lipid parameters, creatinine, and heart rate before and after vitamin D supplementation.

The impact of vitamin D on BP levels is still a topic of debate. Several studies have explored this issue and produced conflicting findings, with some reporting a reduction in BP and others observing no change with vitamin D supplementation [32, 33, 34, 35]. There are several potential explanations for these differences. First of all, the use of vitamin D in an intermittent manner in the majority of these studies [36, 37, 38, 39] may have compromised the analysis, since weekly or monthly doses have different biological impacts than daily administration [35, 40], and are probably less effective [41, 42]. The dosage is another factor. A meta‐analysis by Beveridge et al. [43] claimed that most of the evaluated studies utilized low doses, ranging below the usual dosage range of 1500 to 5000 IU/day [44, 45]. Studies that examine the BP effects of higher daily doses of vitamin D are few [46, 47, 48]. Mirhosseini et al. administered a daily average of 4000 IU and targeted a vitamin D level > 100 ng/mL as part of a retrospective study on 8155 prehypertensive and hypertensive patients. They demonstrated that BP decreased as a result of this intervention [47].

Genetic diversity among subgroups is another important consideration. The majority of studies conducted on this topic have focused on populations of European descent [43]. However, it is possible that the positive impacts of vitamin D may vary among ethnic groups. As a result, conflicting findings in studies examining the effects of vitamin D on patients with T1DM may be attributed to genetic factors. According to a review by Altieri et al. [49], certain polymorphisms, such as CYP27B1 and vitamin D binding protein, have been linked to vitamin D action. Ye et al. proposed that CYP2R1 polymorphisms are associated with a reduced risk of hypertension, independent of vitamin D levels [50]. Additionally, autoimmune disease‐linked vitamin d‐related polymorphisms have been identified in patients with T1DM [51, 52]. Vimaleswaran et al. [53] conducted a genetic analysis of single‐nucleotide polymorphisms (SNP) in a Mendelian randomization study and discovered that the CYP2R1 SNP was independently associated with reduced BP, whereas this association was absent for DHCR7 SNP or the downstream metabolism SNPs (GC and CYP24A1). It is essential to conduct further research on the genetic components to strengthen our findings.

Some authors also proposed that vitamin D may suppress renin through a molecular mechanism [54, 55]. The VDR‐RXR heterodimer cascade could decrease mRNA‐prorenin transcription, hence reducing renin production in juxtaglomerular cells. Furthermore, Beveridge et al. [56] discovered that antihypertensive therapy and other cardiovascular medications, such as ACEI/ARB, interact with vitamin D and inactivate its site of action. This may in some way mask the effect of hormones on BP.

Despite all these, the exact mechanism by which vitamin D reduces BP remains uncertain. Vitamin D might serve as an inverse endocrine regulator for the renin–angiotensin system. Experimental studies propose that cholecalciferol suppresses the renin if the renin–angiotensin system is inappropriately activated [57, 58]. On the other hand, some studies demonstrated the antihypertensive effects of vitamin D to the concurrent use of renin‐angiotensin inhibitors [59, 60]. Vitamin D may have other mechanisms for offering secondary protection to high‐risk hypertensive‐diabetic individuals. According to experimental studies, vitamin D enhances vascular regeneration and re‐endothelialization by promoting the homing of angiogenic myeloid cells to the injured vasculature via stromal cell‐derived factor [61]. It may also regulate the activity of circulating endothelial progenitor cells [62] and macrophages [63], which may have a potential effect on slowing down cellular senescence [64].

Several cross‐sectional and epidemiological studies have established a clear link between vitamin D deficiency and worse cardiovascular outcomes in healthy and high‐risk populations [65, 66]. A recent study by Lieberman et al. highlighted the inverse correlation between vitamin D levels and arterial stiffness, measured using pulse wave velocity, in young patients with T1DM [67]. In terms of treatment, a study of black youth with suboptimal vitamin D levels who were supplemented with 2000 IU/day for 16 weeks showed improvements in carotid‐femoral pulse wave velocity [68]. Similarly, in young adults, vitamin D supplementation at a dose of 300,000 IU per month for 3 months resulted in improved vascular function evaluated by flow‐mediated dilatation [69]. In patients with T2DM with vitamin D levels below 50 nm/L, one dose of 100,000 IU of D2 resulted in improved flow‐mediated dilatation and lower SBP [59]. A meta‐analysis of over 65,000 participants in prospective studies found that a decrease of 25 ng/mL in 25(OH)D levels correlated with a relative risk of 1.03 (95% confidence interval (CI): 1.00–1.06) for cardiovascular diseases [70]. Additionally, multiple studies have linked low vitamin D levels with all‐cause mortality [71, 72]. A meta‐analysis, which comprised 849,412 participants from 73 cohort studies with 66,511 mortality events, discovered that compared to the highest third of baseline 25(OH)D levels, the lowest third had an adjusted relative risk for mortality of 1.35 (95% CI: 1.22–1.49) [73].

Factors such as daily cortisol peak and activation of the sympathetic nervous system in the early hours of the morning can influence morning BP levels, which are measured 2 h after awakening [74, 75, 76]. In a recent review by Muscogiuri et al., an inverse correlation was observed between vitamin D levels and serum cortisol [77]. Vitamin D metabolism shares pathways with adrenal metabolism and vitamin D receptors are present in adrenal cells [77, 78, 79]. A systematic review suggested that elevated morning BP might be connected to hyperactivity of the sympathetic autonomic nervous system [80]. A potential method to validate this hypothesis is to analyze heart rate patterns before and after supplementation during ABPM; however, this aspect was not explored in the current study. Previous research has also identified vitamin D as a regulator of the autonomic nervous system, further supporting this concept [81, 82].

Previous studies have reported contradictory results on the impact of vitamin D supplementation on lipid levels [83, 84, 85]. Bislev et al. conducted a recent randomized placebo‐controlled trial in women with secondary hyperparathyroidism caused by vitamin D insufficiency, which is consistent with our findings. They discovered that cholecalciferol supplementation at a dose of 2800 IU per day for 12 weeks improved HDL levels, but not other lipid parameters [86]. However, another meta‐analysis reported that vitamin D supplementation had slight or insignificant effects on serum lipid profile [84]. Vitamin D may have a positive impact on lipid metabolism by increasing insulin secretion and sensitivity, decreasing hepatic triglyceride synthesis, and increasing serum HDL levels. Furthermore, certain in vitro studies have suggested that vitamin D can inhibit adipocyte differentiation [87] and increase intracellular calcium levels, which may promote lipolysis and inhibit lipogenesis [88].

Several studies have demonstrated the positive impact of vitamin D on glucose control [89, 90, 91], potentially making it a helpful supplementary therapy for patients with T2DM as it may enhance beta cell function [92] and decrease insulin resistance [93]. However, other research has not reported these benefits [94, 95], possibly due to variations in the dosage and duration of vitamin D supplementation.

## 5 Limitations

This study has some limitations. One of the primary limitations is the variation in the dosage of vitamin D across studies, which limits our ability to determine the optimal dosage required for any potential benefits. Secondly, the small number of studies included in our review may limit the generalizability and reliability of our findings. Moreover, most of the studies did not have a control group which also restricted our ability to conduct a meta‐analysis and to provide a quantitative estimate of the effect size.

## 6 Conclusions

Our findings suggest that the effect of vitamin D on BP is inconclusive. Several studies reported no significant changes, while one study reported a reduction in morning BP, and another reported no change in BP in patients with CAN. Further well‐designed, randomized controlled trials with larger sample sizes are needed to fully understand the potential benefits of vitamin D supplementation in this patient population.

## Author Contributions

**Rasoul Ebrahimi:** writing – original draft; writing – review and editing, conceptualization, methodology, software, data curation, resources, formal analysis, validation, investigation, visualization. **Mohammad Mahdi Masouri:** writing – original draft, writing – review and editing, methodology, data curation, resources. **Amir Abbas Salehi Amniyeh Khozani:** writing – original draft, writing – review and editing, methodology, validation, data curation. **Sana Mohammad Soltani:** data curation, writing – review and editing, writing – original draft. **Seyed Aria Nejadghaderi:** writing – original draft, writing – review and editing, conceptualization, supervision, project administration, validation, investigation.

## Ethics Statement

The authors have nothing to report.

## Conflicts of Interest

The authors declare no conflicts of interest.

## Transparency Statement

The lead author Seyed Aria Nejadghaderi affirms that this manuscript is an honest, accurate, and transparent account of the study being reported; that no important aspects of the study have been omitted; and that any discrepancies from the study as planned (and, if relevant, registered) have been explained.

## Supporting information

Supporting information.

## Data Availability

The authors confirm that the data supporting the findings of this study are available within the article and its supplementary materials. The data that supports the findings of this study are available in the supplementary material of this article.
