# Supplementary material for: Effects of Vitamin D Supplementation on Blood Pressure in Patients With Type 1 Diabetes Mellitus: A Systematic Review of Clinical Trials
Source: Health Sci Rep. 2025 Mar 2;8(3):e70524. doi: 10.1002/hsr2.70524 (PMC11872689; doi:10.1002/hsr2.70524)
Supplement: Supplementary file 1 — Supporting information. [file HSR2-8-e70524-s001.docx]

**Table S1.** Search strategy for PubMed, Scopus, Web of Science, Embase and Google Scholar.

| **Database**  **(search date)** | **Step** | **Search strategy** | **Number of results** |
| --- | --- | --- | --- |
| **PubMed**  **(03.28.2024)** | #1 | **((((((((((((((((((((("Diabetes Mellitus, Type 1"[MeSH Terms]) OR ("Diabetes Mellitus, Type 1"[Title/Abstract])) OR ("Insulin-Dependent Diabetes Mellitus"[Title/Abstract])) OR ("Insulin Dependent Diabetes Mellitus"[Title/Abstract])) OR ("Juvenile-Onset Diabetes Mellitus"[Title/Abstract])) OR ("Juvenile Onset Diabetes Mellitus"[Title/Abstract])) OR (IDDM[Title/Abstract])) OR ("Juvenile Onset Diabetes"[Title/Abstract])) OR ("Type 1 Diabetes Mellitus"[Title/Abstract])) OR ("Type 1 Diabetes"[Title/Abstract])) OR ("Diabetes, Type 1"[Title/Abstract])) OR ("Diabetes, Autoimmune"[Title/Abstract])) OR ("Autoimmune Diabetes"[Title/Abstract])) OR ("Brittle Diabetes Mellitus"[Title/Abstract])) OR ("Diabetes Mellitus, Ketosis Prone"[Title/Abstract])) OR ("Ketosis-Prone Diabetes Mellitus"[Title/Abstract])) OR ("Ketosis Prone Diabetes Mellitus"[Title/Abstract])) OR (T1DM[Title/Abstract]) ) OR ("Diabetes Mellitus"[MeSH Terms])) OR ("Diabetes Mellitus"[Title/Abstract])) OR (diabetes[Title/Abstract])) OR (diabet*[Title/Abstract])** | 874677 |
|  | #2 | ((((((((((((((((((((((((((((((((((((((((((((((((((((((((((((((((((((((((((((((((((((((((((((((((("Vitamin D"[MeSH Terms]) OR ("Vitamin D*"[Title/Abstract])) OR ("Vitamin D 2"[Title/Abstract])) OR ("Vitamin D 3"[Title/Abstract])) OR ("25-Hydroxyvitamin D 3"[Title/Abstract])) OR ("Dihydroxyvitamin D*"[Title/Abstract])) OR ("24,25-Dihydroxyvitamin D 3"[MeSH Terms])) OR ("24,25-Dihydroxyvitamin D3"[Title/Abstract])) OR ("(24R)-24,25-Dihydroxyvitamin D3"[Title/Abstract])) OR ("1,25-Dihydroxyvitamin D3"[Title/Abstract])) OR ("1 alpha,25-Dihydroxyvitamin D3"[Title/Abstract])) OR ("1,25-dihydroxy-20-epi-Vitamin D3"[Title/Abstract])) OR ("1,25 dihydroxy 20 epi Vitamin D3"[Title/Abstract])) OR ("D3, 1,25-dihydroxy-20-epi-Vitamin"[Title/Abstract])) OR ("1 alpha, 25-dihydroxy-20-epi-Vitamin D3"[Title/Abstract])) OR ("25-Hydroxyvitamin D 2"[MeSH Terms])) OR ("25-Hydroxyvitamin D 2"[Title/Abstract])) OR ("Hydroxyvitamins D"[Title/Abstract])) OR ("Hydroxyvitamin D*"[Title/Abstract])) OR (“Cholecalciferol”[MeSH Terms])) OR (Cholecalciferol*[Title/Abstract])) OR (colecalciferol[Title/Abstract])) OR ("Hydroxycholecalciferols"[MeSH Terms])) OR (Hydroxycholecalciferol*[Title/Abstract])) OR (hydroxycolecalciferol[Title/Abstract])) OR ("25-Hydroxycholecalciferol"[Title/Abstract])) OR ("25-Hydroxycholecalciferol Monohydrate"[Title/Abstract])) OR ("Dihydroxycholecalciferols"[MeSH Terms])) OR (Dihydroxycholecalciferol*[Title/Abstract])) OR (dihydroxycolecalciferol[Title/Abstract])) OR ("24,25-Dihydroxycholecalciferol"[Title/Abstract])) OR ("24R,25-Dihydroxycholecalciferol"[Title/Abstract])) OR ("1 alpha,25-Dihydroxycholecalciferol"[Title/Abstract])) OR ("1,25-Dihydroxycholecalciferol"[Title/Abstract])) OR (Dihydrocholecalciferol[Title/Abstract])) OR (dihydroxycalciferol[Title/Abstract])) OR ("dihydroxy cholecalciferol"[Title/Abstract])) OR ("dihydroxy vitamin d 3"[Title/Abstract])) OR ("Ergocalciferols"[MeSH Terms])) OR (Ergocalciferol*[Title/Abstract])) OR (Calciferol*[Title/Abstract])) OR (Hydroxyergocalciferol*[Title/Abstract])) OR ("25-Hydroxyergocalciferol"[Title/Abstract])) OR ("25-Hydroxycalciferol"[Title/Abstract])) OR ("25 Hydroxycalciferol"[Title/Abstract])) OR (Doxercalciferol[Title/Abstract])) OR (Colecalciferol[Title/Abstract])) OR (Doxercalciferol[Title/Abstract])) OR (epiergocalciferol[Title/Abstract])) OR ("Dihydrotachysterol"[MeSH Terms])) OR (Dihydrotachysterol*[Title/Abstract])) OR (Tachystin[Title/Abstract])) OR (Dihydrotachysterin[Title/Abstract])) OR (“Calcifediol”[MeSH Terms])) OR (Dedrogyl[Title/Abstract])) OR (Hidroferol[Title/Abstract])) OR (Calderol[Title/Abstract])) OR (Calciol[Title/Abstract])) OR (Calcidiol[Title/Abstract])) OR ("Calcitriol-Nefro"[Title/Abstract])) OR (Ercalcidiol[Title/Abstract])) OR (Calcamine[Title/Abstract])) OR (Alphacalcidol[Title/Abstract])) OR (Alfacalcidol[Title/Abstract])) OR (Paricalcitol[Title/Abstract])) OR (Epicalcitriol[Title/Abstract])) OR (Oxacalcitriol[Title/Abstract])) OR (Calcipotriol[Title/Abstract])) OR (Epicalcitriol[Title/Abstract])) OR (Seocalcitol[Title/Abstract])) OR (Tacalcitol[Title/Abstract])) OR (Oxavitamin[Title/Abstract])) OR (arachitol[Title/Abstract])) OR ("Calcitriol"[MeSH Terms])) OR (Calcijex[Title/Abstract])) OR (Bocatriol[Title/Abstract])) OR (Decostriol[Title/Abstract])) OR ("MC1288"[Title/Abstract])) OR ("MC-1288"[Title/Abstract])) OR (Osteotriol[Title/Abstract])) OR (Renatriol[Title/Abstract])) OR (Rocaltrol[Title/Abstract])) OR (Silkis[Title/Abstract])) OR (Sitriol[Title/Abstract])) OR (Soltriol[Title/Abstract])) OR (Tirocal[Title/Abstract])) OR ("colextra-d3"[Title/Abstract])) OR ("20-epi-1alpha,25-dihydroxycholecaliferol"[Title/Abstract])) OR ("1,25(OH)2-20epi-D3"[Title/Abstract])) OR ("9,10-Secoergosta-5,7,10(19),22-tetraene-3 beta,25-diol"[Title/Abstract])) OR ("(3 beta,5Z,7E)-9,10-Secocholesta-5,7,10(19)-trien-3-ol"[Title/Abstract])) OR ("AT 10"[Title/Abstract])) OR ("AT-10"[Title/Abstract])) OR (AT10[Title/Abstract])) OR ("1, 25-(OH)2D3"[Title/Abstract])) OR ("1,25(OH)2D3"[Title/Abstract]) ) OR (VitD[Title/Abstract])) OR ((("24,25-Dihydroxyvitamin D 3"[Title/Abstract]) OR ("25-Hydroxyvitamin D2"[Title/Abstract])) OR (Calcifediol[Title/Abstract])) | 225617 |
|  | #3 | (((((((((("Clinical Trial"[Publication Type]) OR ("Controlled Clinical Trial"[Publication Type])) OR ("Randomized Controlled Trial"[Publication Type])) OR ("Clinical Trial*"[Title/Abstract])) OR ("controlled clinical trial*"[Title/Abstract])) OR ("controlled trial*"[Title/Abstract])) OR (RCT[Title/Abstract])) OR ("randomized controlled trial*"[Title/Abstract])) OR ("randomized trial"[Title/Abstract])) | 1579869 |
|  | #4 | #1 AND #2 AND #3 | 1402 |
| **Scopus**  **(03.28.2024)** | #1 | ( TITLE-ABS-KEY ( "Type 1 Diabetes Mellitus" ) OR TITLE-ABS-KEY ( "Diabetes Mellitus, Type 1" ) OR TITLE-ABS-KEY ( "Insulin-Dependent Diabetes Mellitus" ) OR TITLE-ABS-KEY ( "Insulin Dependent Diabetes Mellitus" ) OR TITLE-ABS-KEY ( iddm ) OR TITLE-ABS-KEY ( "Juvenile-Onset Diabetes Mellitus" ) OR TITLE-ABS-KEY ( "Juvenile Onset Diabetes Mellitus" ) OR TITLE-ABS-KEY ( "Juvenile Onset Diabetes" ) OR TITLE-ABS-KEY ( "Type 1 Diabetes" ) OR TITLE-ABS-KEY ( "Diabetes, Type 1" ) OR TITLE-ABS-KEY ( "Diabetes, Autoimmune" ) OR TITLE-ABS-KEY ( "Autoimmune Diabetes" ) OR TITLE-ABS-KEY ( "Brittle Diabetes Mellitus" ) OR TITLE-ABS-KEY ( "Diabetes Mellitus, Ketosis Prone" ) OR TITLE-ABS-KEY ( "Ketosis-Prone Diabetes Mellitus" ) OR TITLE-ABS-KEY ( "Ketosis Prone Diabetes Mellitus" ) OR TITLE-ABS-KEY ( t1dm ) OR TITLE-ABS-KEY ( "Diabetes Mellitus" ) OR TITLE-ABS-KEY ( "diabetes" ) OR TITLE-ABS-KEY ( diabet* ) ) | 1303838 |
|  | #2 | ( TITLE-ABS-KEY ( "Vitamin D*" ) OR TITLE-ABS-KEY ( "Vitamin D 2" ) OR TITLE-ABS-KEY ( "Vitamin D 3" ) OR TITLE-ABS-KEY ( "25-Hydroxyvitamin D 3" ) OR TITLE-ABS-KEY ( "Dihydroxyvitamin D*" ) OR TITLE-ABS-KEY ( "24,25-Dihydroxyvitamin D3" ) OR TITLE-ABS-KEY ( "24,25-Dihydroxyvitamin D 3" ) OR TITLE-ABS-KEY ( "(24R)-24,25-Dihydroxyvitamin D3" ) OR TITLE-ABS-KEY ( "1,25-Dihydroxyvitamin D3" ) OR TITLE-ABS-KEY ( "1 alpha,25-Dihydroxyvitamin D3" ) OR TITLE-ABS-KEY ( "1,25-dihydroxy-20-epi-Vitamin D3" ) OR TITLE-ABS-KEY ( "1,25 dihydroxy 20 epi Vitamin D3" ) OR TITLE-ABS-KEY ( "D3, 1,25-dihydroxy-20-epi-Vitamin" ) OR TITLE-ABS-KEY ( "1 alpha, 25-dihydroxy-20-epi-Vitamin D3" ) OR TITLE-ABS-KEY ( "25-Hydroxyvitamin D2" ) OR TITLE-ABS-KEY ( "24,25-Dihydroxyvitamin D 3" ) OR TITLE-ABS-KEY ( "25-Hydroxyvitamin D 2" ) OR TITLE-ABS-KEY ( "Hydroxyvitamin D*" ) OR TITLE-ABS-KEY ( "Cholecalciferol*" ) OR TITLE-ABS-KEY ( "colecalciferol" ) OR TITLE-ABS-KEY ( "Hydroxycholecalciferol*" ) OR TITLE-ABS-KEY ( "hydroxycolecalciferol" ) OR TITLE-ABS-KEY ( "25-Hydroxycholecalciferol" ) OR TITLE-ABS-KEY ( "25-Hydroxycholecalciferol Monohydrate" ) OR TITLE-ABS-KEY ( "Dihydroxycholecalciferol*" ) OR TITLE-ABS-KEY ( "dihydroxycolecalciferol" ) OR TITLE-ABS-KEY ( "24,25-Dihydroxycholecalciferol" ) OR TITLE-ABS-KEY ( "24R,25-Dihydroxycholecalciferol" ) OR TITLE-ABS-KEY ( "1 alpha,25-Dihydroxycholecalciferol" ) OR TITLE-ABS-KEY ( "1,25-Dihydroxycholecalciferol" ) OR TITLE-ABS-KEY ( "Dihydrocholecalciferol" ) OR TITLE-ABS-KEY ( "dihydroxycalciferol" ) OR TITLE-ABS-KEY ( "dihydroxy cholecalciferol" ) OR TITLE-ABS-KEY ( "dihydroxy vitamin d 3" ) OR TITLE-ABS-KEY ( "Ergocalciferol*" ) OR TITLE-ABS-KEY ( calciferol* ) OR TITLE-ABS-KEY ( hydroxyergocalciferol* ) OR TITLE-ABS-KEY ( "25-Hydroxyergocalciferol" ) OR TITLE-ABS-KEY ( "25-Hydroxycalciferol" ) OR TITLE-ABS-KEY ( "25 Hydroxycalciferol" ) OR TITLE-ABS-KEY ( doxercalciferol ) OR TITLE-ABS-KEY ( colecalciferol ) OR TITLE-ABS-KEY ( doxercalciferol ) OR TITLE-ABS-KEY ( epiergocalciferol ) OR TITLE-ABS-KEY ( "Dihydrotachysterol*" ) OR TITLE-ABS-KEY ( tachystin ) OR TITLE-ABS-KEY ( dihydrotachysterin ) OR TITLE-ABS-KEY ( "Calcifediol" ) OR TITLE-ABS-KEY ( dedrogyl ) OR TITLE-ABS-KEY ( hidroferol ) OR TITLE-ABS-KEY ( calderol ) OR TITLE-ABS-KEY ( calciol ) OR TITLE-ABS-KEY ( calcidiol ) OR TITLE-ABS-KEY ( "Calcitriol-Nefro" ) OR TITLE-ABS-KEY ( ercalcidiol ) OR TITLE-ABS-KEY ( calcamine ) OR TITLE-ABS-KEY ( alphacalcidol ) OR TITLE-ABS-KEY ( alfacalcidol ) OR TITLE-ABS-KEY ( paricalcitol ) OR TITLE-ABS-KEY ( epicalcitriol ) OR TITLE-ABS-KEY ( oxacalcitriol ) OR TITLE-ABS-KEY ( calcipotriol ) OR TITLE-ABS-KEY ( epicalcitriol ) OR TITLE-ABS-KEY ( seocalcitol ) OR TITLE-ABS-KEY ( tacalcitol ) OR TITLE-ABS-KEY ( oxavitamin ) OR TITLE-ABS-KEY ( arachitol ) OR TITLE-ABS-KEY ( "Calcitriol" ) OR TITLE-ABS-KEY ( calcijex ) OR TITLE-ABS-KEY ( bocatriol ) OR TITLE-ABS-KEY ( decostriol ) OR TITLE-ABS-KEY ( mc1288 ) OR TITLE-ABS-KEY ( osteotriol ) OR TITLE-ABS-KEY ( renatriol ) OR TITLE-ABS-KEY ( rocaltrol ) OR TITLE-ABS-KEY ( silkis ) OR TITLE-ABS-KEY ( sitriol ) OR TITLE-ABS-KEY ( soltriol ) OR TITLE-ABS-KEY ( tirocal ) OR TITLE-ABS-KEY ( "colextra-d3" ) OR TITLE-ABS-KEY ( "20-epi-1alpha,25-dihydroxycholecaliferol" ) OR TITLE-ABS-KEY ( "1,25(OH)2-20epi-D3" ) OR TITLE-ABS-KEY ( "9,10-Secoergosta-5,7,10(19),22-tetraene-3 beta,25-diol" ) OR TITLE-ABS-KEY ( "(3 beta,5Z,7E)-9,10-Secocholesta-5,7,10(19)-trien-3-ol" ) OR TITLE-ABS-KEY ( "AT-10" ) OR TITLE-ABS-KEY ( "1, 25-(OH)2D3" ) OR TITLE-ABS-KEY ( "1,25(OH)2D3" ) ) | 405780 |
|  | #3 | ( TITLE-ABS-KEY ( "Clinical Trial*" ) OR TITLE-ABS-KEY ( "controlled clinical trial*" ) OR TITLE-ABS-KEY ( "controlled trial*" ) OR TITLE-ABS-KEY ( rct ) OR TITLE-ABS-KEY ( "randomized controlled trial*" ) OR TITLE-ABS-KEY ( "randomized trial" ) ) | 2332499 |
|  | #4 | #1 AND #2 AND #3 | 3871 |
| **Web of Science**  **(03.28.2024)** | #1 | **"Type 1 Diabetes Mellitus" (All Fields) or "Insulin-Dependent Diabetes Mellitus" (All Fields) or "Insulin Dependent Diabetes Mellitus" (All Fields) or "Juvenile-Onset Diabetes Mellitus" (All Fields) or "Juvenile Onset Diabetes Mellitus" (All Fields) or "IDDM" (All Fields) or "Juvenile Onset Diabetes" (All Fields) or "Type 1 Diabetes" (All Fields) or "Autoimmune Diabetes" (All Fields) or "Brittle Diabetes Mellitus" (All Fields) or "Ketosis-Prone Diabetes Mellitus" (All Fields) or "Ketosis Prone Diabetes Mellitus" (All Fields) or "t1dm" (All Fields) or "diabetes" (All Fields) or "diabet*" (All Fields)** | 1350645 |
|  | #2 | **"Vitamin D*" (All Fields) or "Vitamin D 2" (All Fields) or "Vitamin D 3" (All Fields) or "25-Hydroxyvitamin D 3" (All Fields) or "Dihydroxyvitamin D*" (All Fields) or "24,25-Dihydroxyvitamin D3" (All Fields) or "24,25-Dihydroxyvitamin D 3" (All Fields) or "(24R)-24,25-Dihydroxyvitamin D3" (All Fields) or "1 alpha,25-Dihydroxyvitamin D3" (All Fields) or "1,25-dihydroxy-20-epi-Vitamin D3" (All Fields) or "25-Hydroxyvitamin D2" (All Fields) or "24,25-Dihydroxyvitamin D 3" (All Fields) or "25-Hydroxyvitamin D 2" (All Fields) or "Hydroxyvitamin D*" (All Fields) or "Cholecalciferol*" (All Fields) or "colecalciferol" (All Fields) or "Hydroxycholecalciferol*" (All Fields) or "hydroxycolecalciferol" (All Fields) or "25-Hydroxycholecalciferol" (All Fields) or "Dihydroxycholecalciferol*" (All Fields) or "dihydroxycolecalciferol" (All Fields) or "24,25-Dihydroxycholecalciferol" (All Fields) or "24R,25-Dihydroxycholecalciferol" (All Fields) or "1 alpha,25-Dihydroxycholecalciferol" (All Fields) or "1,25-Dihydroxycholecalciferol" (All Fields) or "Dihydrocholecalciferol" (All Fields) or "dihydroxycalciferol" (All Fields) or "dihydroxy cholecalciferol" (All Fields) or "dihydroxy vitamin d 3" (All Fields) or "Ergocalciferol*" (All Fields) or "Calciferol*" (All Fields) or "Hydroxyergocalciferol*" (All Fields) or "25-Hydroxyergocalciferol" (All Fields) or "25-Hydroxycalciferol" (All Fields) or "25 Hydroxycalciferol" (All Fields) or "Doxercalciferol" (All Fields) or "Colecalciferol" (All Fields) or "Doxercalciferol" (All Fields) or "epiergocalciferol" (All Fields) or "Dihydrotachysterol*" (All Fields) or "Tachystin" (All Fields) or "Dihydrotachysterin" (All Fields) or "Calcifediol" (All Fields) or "Dedrogyl" (All Fields) or "Hidroferol" (All Fields) or "Calderol" (All Fields) or "Calciol" (All Fields) or "Calcidiol" (All Fields) or "Calcitriol-Nefro" (All Fields) or "Ercalcidiol" (All Fields) or "Calcamine" (All Fields) or "Alphacalcidol" (All Fields) or "Alfacalcidol" (All Fields) or "Paricalcitol" (All Fields) or "Epicalcitriol" (All Fields) or "Oxacalcitriol" (All Fields) or "Calcipotriol" (All Fields) or "Epicalcitriol" (All Fields) or "Seocalcitol" (All Fields) or "Tacalcitol" (All Fields) or "Oxavitamin" (All Fields) or "arachitol" (All Fields) or "Calcitriol*" (All Fields) or "Calcijex" (All Fields) or "Bocatriol" (All Fields) or "Decostriol" (All Fields) or "MC1288" (All Fields) or "Osteotriol" (All Fields) or "Renatriol" (All Fields) or "Rocaltrol" (All Fields) or "Silkis" (All Fields) or "Sitriol" (All Fields) or "Soltriol" (All Fields) or "Tirocal" (All Fields) or "colextra-d3" (All Fields) or "1, 25-(OH)2D3" (All Fields) or "1,25(OH)2D3" (All Fields)** | 132438 |
|  | #3 | **"Clinical Trial*" (All Fields) or "controlled clinical trial*" (All Fields) or "controlled trial*" (All Fields) or "rct" (All Fields) or "randomized controlled trial*" (All Fields) or "randomized trial" (All Fields)** | 1119943 |
|  | #4 | #1 AND #2 AND #3 | 1975 |
| **Embase**  **(03.28.2024)** | #1 | 'insulin dependent diabetes mellitus'/exp OR 'type 1 diabetes mellitus':ti,ab,kw OR 'insulin-dependent diabetes mellitus':ti,ab,kw OR 'insulin dependent diabetes mellitus':ti,ab,kw OR 'iddm':ti,ab,kw OR 'juvenile-onset diabetes mellitus':ti,ab,kw OR 'juvenile onset diabetes mellitus':ti,ab,kw OR 'juvenile onset diabetes':ti,ab,kw OR 'type 1 diabetes':ti,ab,kw OR 'diabetes, type 1':ti,ab,kw OR 'diabetes, autoimmune':ti,ab,kw OR 'autoimmune diabetes':ti,ab,kw OR 'brittle diabetes mellitus':ti,ab,kw OR 'diabetes mellitus, ketosis prone':ti,ab,kw OR 'ketosis-prone diabetes mellitus':ti,ab,kw OR 'ketosis prone diabetes mellitus':ti,ab,kw OR 't1dm':ti,ab,kw OR 'diabetes mellitus':ti,ab,kw OR 'diabetes':ti,ab,kw OR 'diabet*':ti,ab,kw | 1277273 |
|  | #2 | 'vitamin d*':ti,ab,kw OR 'vitamin d 2':ti,ab,kw OR 'vitamin d 3':ti,ab,kw OR 'dihydroxyvitamin d*':ti,ab,kw OR 'secalciferol'/exp OR '24,25-dihydroxyvitamin d 3':ti,ab,kw OR '24,25-dihydroxyvitamin d3':ti,ab,kw OR '1,25-dihydroxyvitamin d3':ti,ab,kw OR '1 alpha,25-dihydroxyvitamin d3':ti,ab,kw OR '1,25-dihydroxy-20-epi-vitamin d3':ti,ab,kw OR '1,25 dihydroxy 20 epi vitamin d3':ti,ab,kw OR '1 alpha, 25-dihydroxy-20-epi-vitamin d3':ti,ab,kw OR '25 hydroxyergocalciferol'/exp OR '25-hydroxyvitamin d 2':ti,ab,kw OR '25-hydroxyvitamin d2':ti,ab,kw OR '25-hydroxyvitamin d 3':ti,ab,kw OR 'hydroxyvitamin d*':ti,ab,kw OR 'colecalciferol'/exp OR 'cholecalciferol*':ti,ab,kw OR 'hydroxycolecalciferol'/exp OR 'hydroxycolecalciferol':ti,ab,kw OR '25-hydroxycholecalciferol':ti,ab,kw OR '25-hydroxycholecalciferol monohydrate':ti,ab,kw OR 'dihydroxycolecalciferol'/exp OR 'dihydroxycholecalciferol*':ti,ab,kw OR '24,25-dihydroxycholecalciferol':ti,ab,kw OR '24r,25-dihydroxycholecalciferol':ti,ab,kw OR '1 alpha,25-dihydroxycholecalciferol':ti,ab,kw OR '1,25-dihydroxycholecalciferol':ti,ab,kw OR 'dihydrocholecalciferol':ti,ab,kw OR 'dihydroxycalciferol':ti,ab,kw OR 'dihydroxy cholecalciferol':ti,ab,kw OR 'dihydroxy vitamin d 3':ti,ab,kw OR 'ergocalciferol*':ti,ab,kw OR 'ergocalciferol'/exp OR 'calciferol*':ti,ab,kw OR 'hydroxyergocalciferol*':ti,ab,kw OR '25-hydroxyergocalciferol':ti,ab,kw OR '25-hydroxycalciferol':ti,ab,kw OR '25 hydroxycalciferol':ti,ab,kw OR 'colecalciferol':ti,ab,kw OR 'doxercalciferol':ti,ab,kw OR 'epiergocalciferol':ti,ab,kw OR 'dihydrotachysterol'/exp OR 'tachystin':ti,ab,kw OR 'dihydrotachysterin':ti,ab,kw OR 'dihydrotachysterol*':ti,ab,kw OR 'calcifediol':ti,ab,kw OR 'dedrogyl':ti,ab,kw OR 'calderol':ti,ab,kw OR 'calciol':ti,ab,kw OR 'calcidiol':ti,ab,kw OR 'calcitriol nefro':ti,ab,kw OR 'ercalcidiol':ti,ab,kw OR 'calcamine':ti,ab,kw OR 'alphacalcidol':ti,ab,kw OR 'alfacalcidol':ti,ab,kw OR 'paricalcitol':ti,ab,kw OR 'oxacalcitriol':ti,ab,kw OR 'calcipotriol':ti,ab,kw OR 'epicalcitriol':ti,ab,kw OR 'seocalcitol':ti,ab,kw OR 'tacalcitol':ti,ab,kw OR 'oxavitamin':ti,ab,kw OR 'arachitol':ti,ab,kw OR 'calcitriol'/exp OR 'calcitriol':ti,ab,kw OR 'calcijex':ti,ab,kw OR 'bocatriol':ti,ab,kw OR 'decostriol':ti,ab,kw OR 'mc1288':ti,ab,kw OR 'osteotriol':ti,ab,kw OR 'renatriol':ti,ab,kw OR 'rocaltrol':ti,ab,kw OR 'silkis':ti,ab,kw OR 'sitriol':ti,ab,kw OR 'soltriol':ti,ab,kw OR 'tirocal':ti,ab,kw OR 'colextra-d3':ti,ab,kw OR 'at-10':ti,ab,kw OR '1, 25-oh2d3':ti,ab,kw OR '1,25oh2d3':ti,ab,kw | 309184 |
|  | #3 | 'clinical trial*':ti,ab,kw OR 'controlled clinical trial*':ti,ab,kw OR 'controlled trial':ti,ab,kw OR 'rct':ti,ab,kw OR 'randomized controlled trial*':ti,ab,kw OR 'randomized trial':ti,ab,kw OR 'randomized controlled trial'/exp OR 'clinical trial'/exp OR 'controlled clinical trial'/exp | 2507279 |
|  | #4 | #1 AND #2 AND #3 | 2583 |
| **Google Scholar (04.24.2024)** | #1 | "Vitamin D" AND ("Type 1 diabetes" OR "diabetes") | About 1260000 |

**Table S2.** Quality assessment of non-controlled studies.

| **Study ID** | **1** | **2** | **3** | **4** | **5** | **6** | **7** | **8** | **9** | **Overall quality** |
| --- | --- | --- | --- | --- | --- | --- | --- | --- | --- | --- |
| **de Souza et al. 2022 (1)** | Yes | Yes | NA | Yes | Yes | Yes | Yes | Yes | Yes | 8 |
| **De Queiroz et al. 2021 (2)** | Yes | Yes | NA | Yes | Yes | Yes | Yes | Yes | Yes | 8 |
| **Deda et al. 2017 (3)** | Yes | Yes | NA | Yes | Yes | Yes | Yes | Yes | Yes | 8 |
| **Felicio et al. 2017 (4)** | Yes | Yes | NA | Yes | Yes | Yes | Yes | Yes | Yes | 8 |
| **Sliva et al. 2020 (5)** | Yes | Yes | NA | Yes | Yes | Yes | Yes | Yes | Yes | 8 |
| **Felicio et al. 2024 (6)** | Yes | Yes | NA | Yes | Yes | Yes | Yes | Yes | Yes | 8 |

Abbreviations: NA: not available.

1. Was the study question or objective clearly stated?

2. Was the study population clearly and fully described, including a case definition?

3. Were the cases consecutive?

4. Were the subjects comparable?

5. Was the intervention clearly described?

6. Were the outcome measures clearly defined, valid, reliable, and implemented consistently across all study participants?

7. Was the length of follow-up adequate?

8. Were the statistical methods well-described?

9. Were the results well-described?

Overall quality rating: Good: 7-9; Fair: 4-6; and Poor: 0-3

**Table S3.** Quality assessment of controlled intervention studies.

| **Study ID** | **1** | **2** | **3** | **4** | **5** | **6** | **7** | **8** | **9** | **10** | **11** | **12** | **13** | **14** | **Overall quality** |
| --- | --- | --- | --- | --- | --- | --- | --- | --- | --- | --- | --- | --- | --- | --- | --- |
| **Nwosu et al. 2022 (7)** | Yes | Yes | Yes | Yes | Yes | Yes | Yes | Yes | Yes | Yes | Yes | Yes | Yes | Yes | 14 |
| **Joergensen et al. 2014 (8)** | Yes | Yes | NA | Yes | Yes | Yes | Yes | Yes | Yes | Yes | Yes | NA | Yes | Yes | 12 |

Abbreviations: NA: not available.

1. Was the study described as randomized, a randomized trial, a randomized clinical trial, or an RCT?

2. Was the method of randomization adequate (i.e., use of randomly generated assignment)?

3. Was the treatment allocation concealed (so that assignments could not be predicted)?

4. Were study participants and providers blinded to treatment group assignment?

5. Were the people assessing the outcomes blinded to the participants' group assignments?

6. Were the groups similar at baseline on important characteristics that could affect outcomes (e.g., demographics, risk factors, co-morbid conditions)?

7. Was the overall drop-out rate from the study at endpoint 20% or lower of the number allocated to treatment?

8. Was the differential drop-out rate (between treatment groups) at endpoint 15 percentage points or lower?

9. Was there high adherence to the intervention protocols for each treatment group?

10. Were other interventions avoided or similar in the groups (e.g., similar background treatments)?

11. Were outcomes assessed using valid and reliable measures, implemented consistently across all study participants?

12. Did the authors report that the sample size was sufficiently large to be able to detect a difference in the main outcome between groups with at least 80% power?

13. Were outcomes reported or subgroups analyzed prespecified (i.e., identified before analyses were conducted)?

14. Were all randomized participants analyzed in the group to which they were originally assigned, i.e., did they use an intention-to-treat analysis?

Overall quality rating: Good: 11-14; Fair: 7-10; and Poor: 0-6

**References**

1. de Souza A, de Oliveira M, de Lemos GN, da Silva ER, de Souza Í JA, da Silva WM, et al. Health-related quality of life in T1DM patients after high-dose cholecalciferol supplementation: data from a pilot clinical trial. Diabetol Metab Syndr. 2022;14(1):46.

2. de Queiroz NNM, de Melo FTC, de Souza Resende F, Janaú LC, de Souza Neto NJK, de Lemos MN, et al. High-dose Cholecalciferol Supplementation Reducing Morning Blood Pressure in Normotensive DM1 Patients. Curr Diabetes Rev. 2021;17(3):378-86.

3. Deda L, Yeshayahu Y, Sud S, Cuerden M, Cherney DZ, Sochett EB, et al. Improvements in peripheral vascular function with vitamin D treatment in deficient adolescents with type 1 diabetes. Pediatr Diabetes. 2018;19(3):457-63.

4. Felício JS, de Oliveira AF, Peixoto AS, de Souza A, Abrahão Neto JF, de Melo FTC, et al. Albuminuria Reduction after High Dose of Vitamin D in Patients with Type 1 Diabetes Mellitus: A Pilot Study. Front Endocrinol (Lausanne). 2017;8:199.

5. Silva LSD, de Queiroz NNM, de Melo FTC, Abrahão Neto JF, Janaú LC, de Souza Neto NJK, et al. Improvement in Cardiovascular Autonomic Neuropathy After High-Dose Vitamin D Supplementation in Patients With Type 1 Diabetes. Front Endocrinol (Lausanne). 2020;11:605681.

6. Felício J, Moraes L, Lemos G, Souza Í, Vieira G, Silva L, et al. High dose cholecalciferol supplementation causing morning blood pressure reduction in patients with type 1 diabetes mellitus and cardiovascular autonomic neuropathy. Sci Rep. 2024;14(1):6374.

7. Nwosu BU, Parajuli S, Jasmin G, Fleshman J, Sharma RB, Alonso LC, et al. Ergocalciferol in New-onset Type 1 Diabetes: A Randomized Controlled Trial. J Endocr Soc. 2022;6(1):bvab179.

8. Joergensen C, Tarnow L, Goetze JP, Rossing P. Vitamin D analogue therapy, cardiovascular risk and kidney function in people with Type 1 diabetes mellitus and diabetic nephropathy: a randomized trial. Diabet Med. 2015;32(3):374-81.
